# Editorial: Neuroinflammation, metabolism, and psychiatric disorders

**DOI:** 10.3389/fpsyt.2022.1060948

**Published:** 2022-10-18

**Authors:** Zachary Freyberg, Marion Leboyer, Brenda W. J. H. Penninx, Ryan W. Logan

**Affiliations:** ^1^Department of Psychiatry, University of Pittsburgh, Pittsburgh, PA, United States; ^2^Department of Cell Biology, University of Pittsburgh, Pittsburgh, PA, United States; ^3^Université Paris Est Créteil (UPEC), INSERM, IMRB, Translational Neuropsychiatry Laboratory, AP-HP, DMU IMPACT, FHU ADAPT (Hopital Henri Mondor), Créteil, France; ^4^Fondation FondaMental, Hôpital Albert-Chenevier, Créteil, France; ^5^Department of Psychiatry, Amsterdam UMC Location Vrije Universiteit Amsterdam, Amsterdam Neuroscience, Mood, Anxiety, Psychosis, Sleep and Stress Program, Amsterdam, Netherlands; ^6^Department of Pharmacology and Experimental Therapeutics, Boston University School of Medicine, Boston, MA, United States; ^7^Center for Systems Neuroscience, Boston University, Boston, MA, United States; ^8^Genome Science Institute, Boston University School of Medicine, Boston, MA, United States

**Keywords:** neuroinflammation, metabolism, autism spectrum disorder, schizophrenia, ADHD, major depressive disorder, anxiety disorder, metabolic syndrome

Growing evidence suggests that immunologic dysregulation is an important feature of both metabolic disease and psychiatric disorders including schizophrenia, major depressive disorder (MDD), bipolar disorder, anxiety disorders, anorexia nervosa, and neurodevelopmental disorders such as autism and attention-deficit/hyperactivity disorder (ADHD) (1–8). Indeed, chronic inflammation increases vulnerability to the onset of both psychiatric illnesses as well as metabolic syndrome (9, 10). However, the nature of the relationships between the psychiatric, inflammatory, and metabolic disturbances remains unclear. In this Research Topic, our goal was to pave the way for an improved understanding of these complex relationships. Consequently, we present a wide spectrum of original research and review papers that aim to: (1) draw attention to existing gaps in knowledge concerning the interplay of inflammation, metabolic dysfunction, and psychiatric disorders; (2) explore the bidirectional relationships between inflammation, metabolic disturbances, and psychiatric illness; and (3) discuss how inflammatory mechanisms are targeted to treat both metabolic dysfunction and psychiatric disorders.

Our Research Topic features the following original research studies:

DiCarlo et al. offered a novel mechanism for autism spectrum disorder (ASD) pathophysiology associated with dopamine transporter (DAT) function and its relationships to metabolism. The authors demonstrated that altered DAT function in the context of a genetic variant associated with ASD changed dopaminergic neurotransmission and had significant impacts on metabolism, glucose handling, and the oral microbiome.

Lin and Huang examined the relationships between MDD and immune system activation. They discovered that MDD patients exhibited differences in the expression of markers of the innate immune response compared to non-depressed subjects. These results therefore suggest that altered regulation of innate immune activation may play a role in MDD pathophysiology.

Efthymiou et al. linked changes in gait to biomarkers of inflammatory states and metabolic syndrome in antipsychotic drug-treated individuals with psychosis. They showed that gait alterations were associated with metabolic syndrome in these patients. This raises the possibility that changes in gait can predict onset of metabolic syndrome in people with psychosis. Similarly, these data raise the intriguing possibility that adverse effects of some antipsychotic drugs may impact both metabolism and gait.

Chand et al. characterized sphingosine-1-phosphate receptor-1 (S1PR1), a gene expressed in astrocytes and microglia. The authors showed that S1PR1 was significantly upregulated in the dorsolateral prefrontal cortex of patients with one subtype schizophrenia while remaining unaffected in another subtype of the illness.

Pavlinek et al. showed that acute interferon-γ exposure significantly elevated gene expression of neuroimmune factors in human neuron iPSC-derived neurons, including major histocompatibility complex I and complement component 4A (C4A), while downregulating synapsins. Intriguingly, these C4A findings are consistent with C4's association with heightened schizophrenia risk (11).

Jiang et al. conducted metabolic profiling that compared cohorts of individuals with schizophrenia with or without cognitive impairment vs. matched unaffected comparison subjects. The authors found several differentially expressed metabolites in subjects with both schizophrenia and cognitive impairment including those associated with amino acid metabolism and the Krebs cycle, an important component of aerobic respiration.

Barko et al. examined microglial biology by RNA-sequencing TMEM119^+^ microglia and found substantial brain region-specific expression differences. Microglia in midbrain were enriched in transcripts similar to disease-associated or immune-surveillant microglia, while prefrontal cortical microglia showed enrichment in synapse-associated pathways. In contrast, striatal microglia exhibited enrichment in microtubule polymerization-related pathways. There were also sex differences in microglial transcriptomes across all brain regions assayed, suggesting region and sex are crucial determinants of microglial signaling pathways and function. Consequently, these results may provide a novel mechanism that explains the brain region- and sex-specific differences in microglia-driven inflammation that contribute to the pathophysiology of psychiatric disorders.

Gustafsson et al. investigated alterations in polyunsaturated fatty acids alongside systemic inflammation associated with ADHD during pregnancy *via* a cross-sectional analytical observational research study in human subjects. The authors demonstrated that subjects with heightened ADHD symptoms possessed increases in the ratio of omega-6 to omega-3 polyunsaturated fatty acids as well as elevated levels of TNF-α, a pro-inflammatory cytokine. These findings suggest cause-and-effect relationships between factors such that there is an association between ADHD and changes in fatty acid metabolism that modify inflammatory states.

Freff et al. explored the relationships between anorexia nervosa (AN) and enhanced inflammation, showing increased expression of chemokine receptors CCR4, CXCR3, and CXCR4 on CD4^+^ T-cells in AN vs. controls. Additionally, T-cell CXCR4 expression predicted body composition in adolescents. These data suggest important links between chemokine receptor expression, inflammatory states and AN, offering a new mechanism for AN pathogenesis.

We also feature the following reviews:

Tateishi et al. reviewed the therapeutic mechanisms underlying repetitive transcranial magnetic stimulation for treatment of cognitive dysfunction in depression, focusing on potential roles that neuroinflammation plays in these processes.

Rahimian et al. reviewed the involvement of microglia in the neuroinflammatory processes that contribute to the pathogenesis of MDD.

## Author contributions

All authors listed have made a substantial, direct, and intellectual contribution to the work and approved it for publication.

## Funding

This work was supported by the following grants: National Institutes of Health R01 DK124219 (ZF), R21 AG068607 (ZF), R21 DA052419 (ZF and RL), R21 AA028800 (ZF and RL), R01 DA051390 (RL), R01 HL150432 (RL), and R33 DA041872 (RL), as well as by the Department of Defense PR210207 (ZF).

## Conflict of interest

The authors declare that the research was conducted in the absence of any commercial or financial relationships that could be construed as a potential conflict of interest.

## Publisher's note

All claims expressed in this article are solely those of the authors and do not necessarily represent those of their affiliated organizations, or those of the publisher, the editors and the reviewers. Any product that may be evaluated in this article, or claim that may be made by its manufacturer, is not guaranteed or endorsed by the publisher.
